# Endovascular treatment of penetrating vascular injuries

**DOI:** 10.1093/jscr/rjab486

**Published:** 2021-11-11

**Authors:** Roozbeh Cheraghali, Javad Salimi, Zahra Omrani

**Affiliations:** Vascular and Endovascular Surgery, Tehran University of Medical Sciences (TUMS), Tehran, Iran; Vascular and Endovascular Surgery, Liver Transplantation Program, Tehran University of Medical Sciences, Tehran, Iran; Department of Surgery, Iran University of Medical Sciences (IUMS), Tehran, Iran

## Abstract

Endovascular treatment of vascular injuries has resulted in reduced operating time, blood loss, hospital mortality and sepsis. The purpose of this study was to evaluate the success and complication rate of the endovascular management of penetrating peripheral vascular injuries during 5 years. In this observational study, the clinical records and imaging features of 22 penetrating trauma injuries of 276 penetrating vascular trauma patients (8%), which were repaired using endovascular stent-grafts or coil embolization, between April 2013 and August 2018, included in the study. The median age of patients was 43 years (Range, 20–78 years). There were 17 stab wounds (77.3%), 2 shotgun war remnants (9.1%) and 2 iatrogenic post-surgical lesions. Eleven stent-grafts (50%) and nine coil embolizations (40.9%) were deployed. Endovascular interventions in the management of peripheral vascular injuries can be efficient in definitive repair, damage control and hemorrhage control in severely ill trauma patients.

## INTRODUCTION

Using angiography in the diagnosis of vascular injuries over the years has evolved into the endovascular methods of treatment [1]. It has resulted in reduced operating time, blood loss, hospital mortality and sepsis [2–4]. Major findings after penetrating vascular injuries include occlusion, extravasation, pseudoaneurysm (PSA) and arteriovenous fistula (AVF). Intra-luminal filling defects such as thrombi and intimal flaps can also be detected. Minor findings include luminal narrowing, focal widening of the lumen, arterial deviation and slow flow that may be encountered in the calf due to high compartment pressures [1, 5].

With the development of endovascular techniques, the management of peripheral vascular injuries with covered stents or coil embolization has become more accepted by trauma care physicians [6].

It is indicated in a patient with ‘hard signs’ of vascular injury, which include pulsatile or expanding hematoma, diminished or absent pulses, bruit or thrill, critical limb ischemia or active hemorrhage [7].

Embolization for traumatic vascular injury was first declared in the early 1970s, with the reports by Rosch and Dotter [8] in 1972 and Bookstein and Goldstein [9] 1 year later.

Closure of the pseudoaneurysm, AVF or healing of the dissection with preservation of the original artery is the basic goal of treatment. The only definite contraindication to endovascular repair of an injury is the inability to cross a lesion with a wire unless the aim of the procedure is hemorrhage control with embolization [10]. Vessel sacrifice is sometimes indicated, however, as the last decision [11].

Covered stents allow treatment of PSA, AVF and other injuries in the renal, iliac, subclavian and axillary arteries and other nonexpendable vessels [12–14].

The purpose of this study was to evaluate the success and complication rate of the endovascular management of penetrating peripheral vascular injuries during 5 years.

## CASE SERIES

### Methods

The clinical records and imaging features of all 22 penetrating trauma injuries of 276 penetrating vascular trauma patients (8%), which were repaired using endovascular stent-grafts or coil embolization, between April 2013 and August 2018, included in this observational study. Surgically treated patients and patients with blunt non-penetrating injuries were excluded from the study. There were 18 males (81.8%) and 4 females (4.2%). The median age of patients was 43 years (Range, 20–78 years). Eighteen stab wounds, two shotgun war remnants and two iatrogenic postsurgical peripheral damages were treated (Table 1). Sina Hospital is one of the important centers in Iran with the capability of 24-hour emergent endovascular interventions of neurologic, cardiac and peripheral vascular injuries. Institutional review board approval was obtained for this retrospective project.

**Table 1:** Summary of cases

Patient No.	Sex/Age	Lesion type	Lesion location	Intervention CE/SG	Trauma-intervention interval	Follow-up (months)
1	F,58	PA	posterior Tibialis artery	1 SG 4*40 mm	5 days	4.0
2	M,23	PA	Maxillary artery	3 CE 3 mm	2 months	20.0
3	M,37	PA	posterior Tibialis artery	1 SG 5*60 mm	4 days	22.0
4	M,22	PA	Maxillary artery	5 CE (4)3 mm (1)5 mm	10 days	24.0
5	F,57	PA	Anterior Tibialis artery	3 CE (2) 5 mm (1)3 mm	1 month	20.0
6	M,78	PA	Superficial femoral artery	1 SG 8*60 mm	3 days	27.0
7	M,50	AVF	Posterior Tibialis artery	2 SG 5*60 mm 4*40 mm	22 days	3.0
8	M,40	PA/AVF	Deep femoral artery and vein	1 SG 6*40 mm	1 month	23
9	M,53	PA	Proximal of left external carotid artery	1 SG 7*40 mm 2 CE 8 mm 10 mm	3 days	25.0
10	M,47	PA	Superficial Femoral artery	2 SG 7*60 mm 7*40 mm	2 days	22.0
11	F,38	PA	Tibioproneal trunk	2 SG 5*40 mm 5*60 mm	2 months	21.0
12	M,42	PA/AVF	Posterior Tibialis artery	1 SG 4*40 mm	12 days	20.0
13	M,75	PA	Posterior Tibialis artery	1 SG 4*40 mm	7 days	27.0
14	M,27	PA	Temporal artery	2 CE 2 mm 3 mm	21 days	23.0
15	M,30	PA	Axillary artery	1 CE 5 mm	2 days	22.0
16	M,28	PA/AVF	Internal iliac artery	2 CE 5 mm	2 days	27.0
17	M,28	PA	Lumbar artery	2 CE 2 mm	4 days	25.0
18	M,20	PA	Internal pudendal Bulbar artery	1 CE 2 mm	18 days	19.0
19	M,55	AVF	posterior Tibialis artery	2SG 4–40 5–40	25 years	27.0
20	M,61	PA	Thoracic aorta	SG 28–80	25 years	36.0
21	M,44	PA	Common iliac artery	SG 10–60	2 days	24.0
22	F,45	PA	Deep femoral artery	2 CE 4 mm 5 mm	8 days	4.0

The clinical information was accessible in the electronic database of the Sina Hospital including demographics, mechanism of trauma, clinical presentation, topography, the morphology of lesions, endovascular techniques used and angiographic images, complications, trauma-intervention interval and clinical results.

Two types of endovascular stent grafts were used: Fluency endovascular stent-grafts and Cook for thoracic endovascular aortic repair (TEVAR) procedures. Gore and Viabahn stent-grafts do not exist in Iran due to economical sanctions.

The suspected patients were referred to us and selective angiography was performed to confirm the diagnosis. All the procedures were conducted in the angiography unit of Sina Hospital, Tehran, Iran. Percutaneous or open femoral access was obtained for extremities, neck, aorta and pelvis trauma. Technical success was defined as the establishment of in-line flow at the end of the endovascular procedure as determined by the completion angiogram. All procedures were conducted by one vascular and endovascular surgeon.

In 14 cases, stent-graft deployment or coil embolization was performed via a retrograde femoral approach. In 8 cases with Tibioproneal trunk or posterior Tibialis pseudoaneurysms or AVF, antegrade femoral approaches were preferred.

In 2 cases with deep femoral AVFs and pseudoaneurysm, a flexible 7 Fr, 45-cm-long introducer sheath was placed across the aortic bifurcation to the contra-lateral side by the cross-over technique. In only one case with deep femoral artery and vein AVF, coil embolization could not exclude the damaged lesion so a 6*40 mm stent-graft was successfully deployed. Details of stent grafts and coils are listed in Table 1.

All patients were given a 5000-IU heparin bolus, intra-arterially, during the procedure. Adjunctive therapy included low molecular weight heparin (Fraxiparine, Sanofi Pharma, Paris, France) for 3 days (two doses a day, 0.4 ml per dose, subcutaneously administered), 75-mg clopidogrel per day for 1 month and 300-mg acetylsalicylic acid per day for whole life.

The follow-up protocol included clinical examination and color Doppler ultrasonography the day after the procedure, and at 1, 3, 6, 9 and 12 months, and then annually, unless the patient had any complaints. According to the site of injury, intravenous or intra-arterial digital subtraction angiography or computed tomography angiography were conducted in selected patients who had complaints or positive color Doppler ultrasonography findings. The follow-up period was 0–36 months (Mean: 21.13, SD = 7.98).

SPSSV.18 was used for data analyzing. Mean, median, standard deviation, frequency and percentile were declared for quantitative and qualitative variables, respectively.

## RESULTS

From April 2013 to August 2018, 276 penetrating vascular trauma cases were admitted to our hospital. Of these, 22 patients (8%) underwent endovascular intervention. There were 17 stab wounds (77.3%), 2 shotgun war remnants (9.1%) and 2 iatrogenic post-surgical lesions. Iatrogenic injuries occurred after 2 femoral bone fracture fixations and one discectomy that the deep femoral artery and right common iliac artery were the affected sites of injury. Trauma-intervention interval varied from 2 days to 25 years in shotgun war remnant patients.

**Figure 1:**
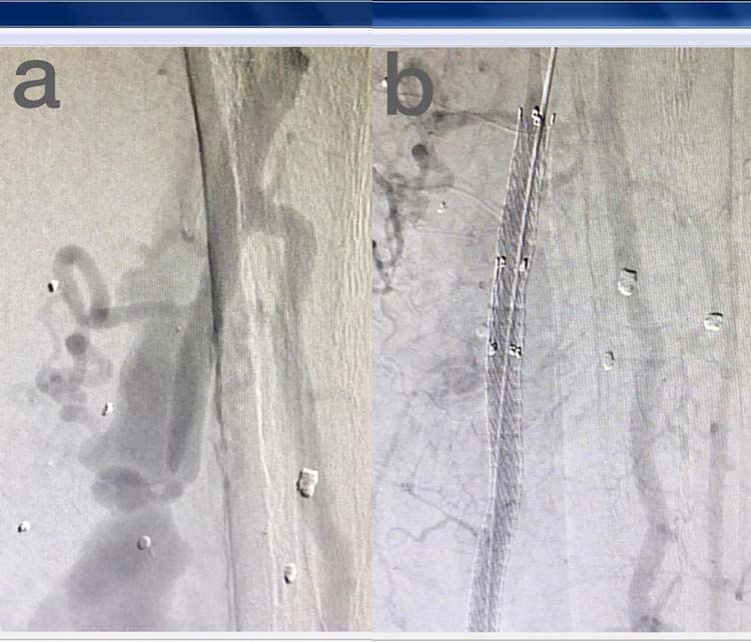
(**a**) Posterior Tibialis artery before intervention, (**b**) after endovascular stent graft deployment.

**Figure 2:**
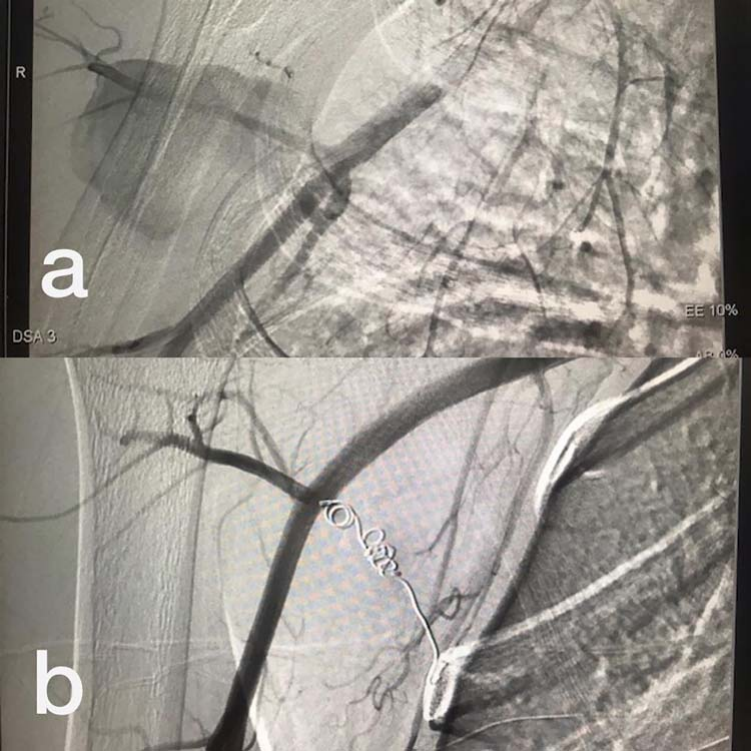
(**a**) Branch of Axillary artery before intervention, (**b**) after coil embolization.

**Figure 3:**
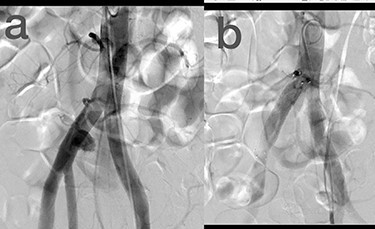
(**a**) Common iliac artery before intervention, (**b**) after stent-graft deployment.

**Figure 4:**
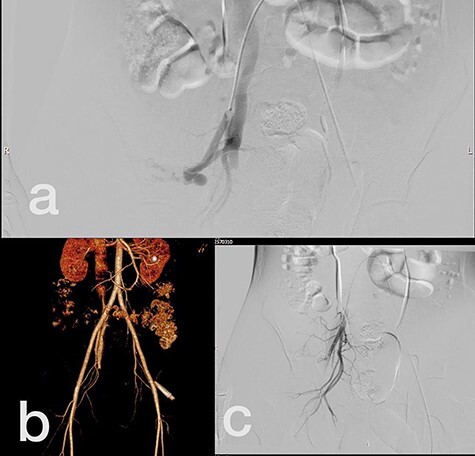
(**a**) Internal iliac artery before intervention, (**b** and **c**) after coil embolization.

Sites of injury included 6 Posterior Tibialis arteries (27.2%) (Fig. 1), 2 deep femoral arteries (9.09%), 2 Superficial femoral arteries (9.09%) and 2 maxillary arteries (9.09%). We had only one injured artery (4.54%) in each of the following sites: internal pudendal a., Axillary a. (Fig. 2), Proneal a., Lumbar a., External carotid a., Anterior Tibialis a., common iliac a. (Fig. 3), temporal a., Thoracic aorta and Internal iliac artery (Fig. 4).

Eleven stent-grafts (50%) and nine coil embolizations (40.9%) were deployed. In 2 patients (9.1%) with proximal external carotid pseudoaneurysm (PA) and deep femoral artery AVF and PA, both stent graft and coil embolization were used. Initially coil embolization was tried for the DFA injury but the lesions were not excluded, so a 6*40 mm stent-graft was deployed with acceptable outcomes. Successful endovascular interventions without any major or minor complications were performed in 14 patients (63.6%). The mean follow-up time was 21.13 (SD = 7.98). Thrombosis (13.6%) and transient rise of creatinine (9.1%) each happened in 3 and 2 patients, respectively. We had one access site hematoma (4.5%), one non-flow limiting disecssion (4.5%) and one small access site pseudoaneurysm (4.5%).

The total success rate of endovascular treatment in this study was 100%. Of 13 successful stent-grafts, thrombosis occurred after 3–4 months in three cases (23.1%) with posterior Tibialis artery injuries. The median patency of stent-grafts was 22 months (Range, 3–36 months).

## DISCUSSION

It is serious to be aware of the potential for a delayed hemorrhage in the setting of penetrating trauma [5]. There can be a latent period, from weeks to even years, between the trauma and the life-threatening hemorrhage. This may be due to pseudoaneurysm formation with the initial trauma, delayed expansion and rupture [7]. Two patients with a history of a shotgun 20 years ago, referred to us by pseudoaneurysm of the thoracic aorta and AVF in the posterior Tibialis artery and vein. Stent-graft deployment was performed successfully for both of them with no procedure-related complication after 36 and 27 months, respectively.

Published technical and clinical success rates of endovascular treatment of arterial injury in the extremities range from 80 to 100% with relatively few complications and as high as 83–100% in the treatment of solid organ arterial injury [7]. The success rate in this study was 100%. Thrombosis occurred in 2 patients (9.1%) and both of them happened in the below-knee arteries (posterior Tibialis a.) which might be ligated if open surgery was conducted for these patients.

Patient selection for angiography is based on different factors such as adjacency, clinical evaluation and physical examination by the trauma team, hemodynamic stability and blunt versus penetrating trauma [1].

Most available coils have thrombogenic fibers attached to their metal frame. Consideration of ongoing coagulopathy in many trauma patients is one of the reasons to use multiple agents. Sometimes, occlusion with coils might be partially effective or lead to delayed bleeding in a coagulopathic patient [1]. In this series, we did not have any delayed bleeding in our coil embolization patients during the follow-up period.

The risks of trauma angiography and embolization are the same as the other angiographic procedures. The most common of these potential complications include contrast reactions and vascular injury, as one access site hematoma (4.5%), one non-flow limiting disecssion (4.5%) and one small access site pseudoaneurysm (4.5%) were detected in our study population.

In a prospective study of 100 consecutive patients undergoing embolization for bleeding in the abdomen and pelvis, 5 patients (5%) developed contrast nephropathy, with creatinine returning to baseline within 5 days in all patients [12]. In our study, 2 patients (9.1%) had transient creatinine rise, returning to baseline within 4 days.

Embolization can lead to a greater area of tissue loss than expected at the time of embolization. However, the greatest fear is non-target embolization into distant or adjacent vessels [1]. Potential complications of covered stent placement include stent occlusion, deformation and kinking and loss of vessel branches after stent placement. Although rare, covered stents have the probability of getting infected in the presence of bacteremia and sepsis [13]. In this study period, there was no coil or stent-related complication except one non-flow limiting disecssion (4.5%) and one small access site pseudoaneurysm (4.5%).

Adequate sizing is also critical with stent-grafts. Stent-graft under-sizing will result in an endoleak due to inadequate sealing of the vessel wall [13]. Therefore, 10–20% over sizing was used for stent-graft deployment.

Information about the durability of carotid and vertebral injuries endovascular interventions is rare, but available data show that selective use of these techniques is safe and durable. Du Toit *et al.* examined a series of 19 zones 1 and 3 penetrating carotid injuries treated with stent-grafts over 10.5 years. The technical success rate was 100% with one stroke within 30 days of the procedure [15].

The first report of TEVAR was published in the 1990s and this has now become the technique that is most used, consisting of deployment of an endograft from peripheral access [16]. This method of treatment stands out, especially for traumatic aortic injuries and complicated acute thoracic aorta dissection, because of the lower morbidity and mortality compared with traditional surgical repair [17]. We had a 61-year-old male with pseudoaneurysm in his thoracic aorta, who underwent TEVAR without procedure related complications after 36 months.

Diego *et al.* studied the outcomes of endovascular treatment of 36 traumatic carotid lesions. Their success rate in endovascular carotid treatment was 97.2%. In this study, only one proximal external carotid damage was successfully treated by a stent-graft. External carotid artery embolization was very effective for the treatment of bleeding in their series and epistaxis was the most frequent type of hemorrhage [11].

In conclusion, endovascular interventions in the management of peripheral vascular injuries can be efficient in definitive repair, damage control and hemorrhage control in severely ill trauma patients. Embolization should be performed early in the control of arterial bleeding before severe coagulopathy develops. However, criteria for the best decision in each case must be individualized.

## AVAILABILITY OF DATA AND MATERIALS

Patients data are available in the electronic database of Sina Hospital of Tehran University of Medical sciences.

## CONFLICT OF INTEREST STATEMENT

None declared.

## FUNDING

None.
